# Group-theoretic analysis of symmetry-preserving deployable structures and metamaterials

**DOI:** 10.1098/rsta.2023.0352

**Published:** 2024-09-09

**Authors:** Gregory S. Chirikjian

**Affiliations:** ^1^ Department of Mechanical Engineering, National University of Singapore, Singapore, Singapore

**Keywords:** deployable Structure, metamaterial, origami

## Abstract

Many deployable structures in nature, as well as human-made mechanisms, preserve symmetry as their configurations evolve. Examples in nature include blooming flowers, dilation of the iris within the human eye, viral capsid maturation and molecular and bacterial motors. Engineered examples include opening umbrellas, elongating scissor jacks, variable apertures in cameras, expanding Hoberman spheres and some kinds of morphing origami structures. In these cases, the structures either preserve a discrete symmetry group or are described as an evolution from one discrete symmetry group to another of the same type as the structure deploys. Likewise, elastic metamaterials built from lattice structures can also preserve symmetry type while passively deforming and changing lattice parameters. A mathematical formulation of such transitions/deployments is articulated here. It is shown that if X is Euclidean space, G is a continuous group of motions of Euclidean space and Γ is the type of the discrete subgroup of G describing the symmetries of the deploying structure, then the symmetry of the evolving structure can be described by time-dependent subgroups of G of the form $\Gamma_{\alpha_{t}}:=\alpha_{t}\Gamma \alpha_{t}^{-1}$ , where $\alpha_{t}$ is a time-dependent affine transformation. Then, instead of considering the whole structure in X , a ‘sector’ of it that lives in the orbit space $\Gamma_{\alpha_{t}}\backslash X$ can be considered at each instant in time, and instead of considering all motions in G , only representatives from right cosets in the space $\Gamma_{\alpha_{t}}\backslash G$ need to be considered.

This article is part of the theme issue 'Current developments in elastic and acoustic metamaterials science (Part 1)'.

## 1. Introduction

Beautiful and efficient deployable structures have existed in nature for eons. Bacteriophages and other viruses have characteristic symmetrical heads that expand to fit genetic material. Rotary molecular motors such as ATP synthase clearly have symmetry as they undergo conformational changes. One can even argue that locomotion proteins such as actin–myosine constitute a form of symmetry-preserving deployment. For pointers to this immense literature see [1–6]. At the macroscopic scale, the blooming of a flower or the reaction to light in the iris of an animal eye continue to be sources of wonder.

Such time-evolving symmetries can be observed in the engineering world as well. Objects as familiar as an umbrella, a scissor jack or the diaphragm in a variable aperture camera preserve finite symmetry type during deployment. More complex structures such as the Hoberman spheres have entertained children and adults alike over the past few decades.

The mathematical concept of a ‘group’ is a fundamental concept used frequently in abstract algebra, geometry and topology. This concept is roughly 200 years old, and has been used extensively in crystallography, quantum mechanics and particle physics. A group consists of a set and an operation for recombining pairs of elements in the set to produce another element in the set. The group operation must be associative, every element must have an inverse and the set must have an identity element. The underlying set can be finite (such as the set of permutations on n letters), discrete but countably infinite (such as the integers) or it can be a continuous manifold consisting of an uncountably infinite number of elements (such as motions of Euclidean space). Groups can be constructed to ‘act on’ other sets by scrambling the elements of the other set or moving them from one location to another. According to Noether’s theorem, every conservation law of a physical system corresponds to a continuous (Lie) group.

In physics, specific fixed symmetry groups are used to describe preserved quantities. In contrast, a basic premise of this article is that the broader concept of symmetry ‘type’ is most relevant in describing what is preserved in deployable mechanisms and metamaterials. For example, the integers, ℤ , mark discrete static points on the real line and the group of motions of the integers equipped with the operation of addition, (ℤ,+) , acts by sliding a copy of the integers by whole numbers so as to coincide with the original arrangement. But if the integers are all multiplied by a non-integer number, a , the resulting scaled version of the integers aℤ , have a discrete symmetry group (aℤ,+) , which is different from the original but of the same type. For example, at any state of extension an accordion-like origami structure or scissor jack has the same basic symmetry type, in which there is a different specific group for each level of extension or expansion.

Whereas in physics groups are static objects that reduce the complexity of describing dynamic phenomena, in this article, it is shown that the evolution of groups within classes of the same type is the natural tool to describe both deployable mechanisms and some kinds of metamaterials.

The remainder of this introduction reviews mathematical concepts in an elementary and graphical way as a reference for the reader. More advanced concepts are encountered later in the article.

### (a) Lattice deployable structures and metamaterials

**Figure 1.**
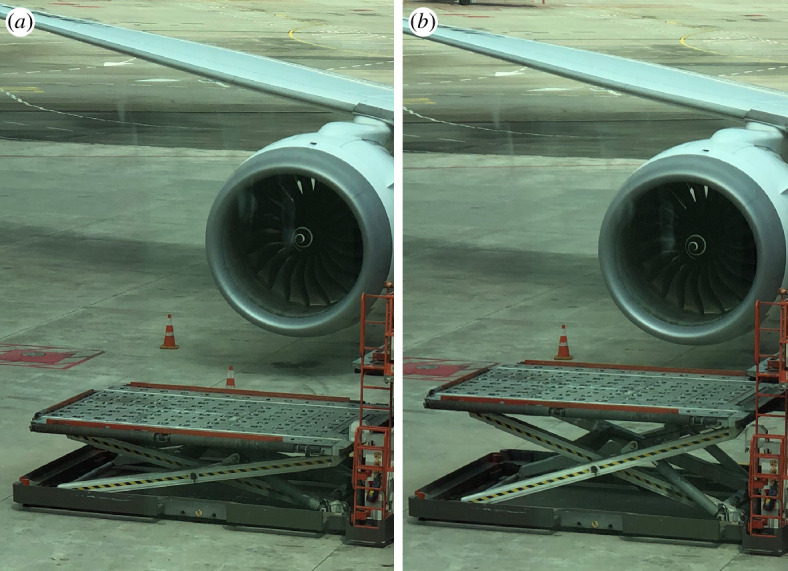
A single-stage scissor-jack mechanism used to load an aeroplane. (*a*,*b*) Two different values of the height of the scissor jack platform, ‘a’.

**Figure 2.**
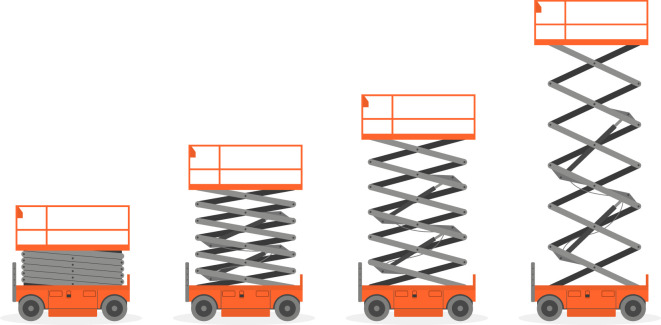
Scissor-jack mechanisms with (aℤ,+) symmetry type. Multiple basic units of the kind in the previous figure are assembled by group action (*source*: shutterstock.com with purchased license).

As a mundane yet explanatory example of this, consider the photograph of a scissor-jack lift shown in figure 1 taken by the author while waiting at an airport during the writing of this article. The mechanism expands in order that supplies can be loaded on the aeroplane. When the extension to greater heights is required (e.g. to enable workers to paint a building or to wash windows), then the basic units in figure 1 can be repeated, as shown in the commercially available system shown in figure 2 which consists of six repeats. The stacking of units can be thought of as a group action of the integers that takes each unit and ‘adds’ them on top of each other. That describes the basic assemblage, but does not address the extension of the mechanism, which is a process of moving between copies of the integers scaled in different ways.

The groups $\left(a_{1}\mathbb{Z},+\right)$ and $\left(a_{2}\mathbb{Z},+\right)$ are different if $a_{1}\neq a_{2}$ are two positive real numbers, but they are of the same type. Algebraically these are the same abstract group, but practically they are different. In solid-state physics, a crystal usually has a definitive lattice symmetry group, and while it is of a standard type, the lattice parameters are considered fixed. In contrast, for deployable/morphable structures it is often the case that only the type of discrete symmetry is preserved and not a specific group. As an example of this, consider a garden trellis. This two-dimensional structure may be purchased at a hardware store in its compressed state, and expanded when installed in a yard. In the compressed and expanded states, the trellis has an abstract two-dimensional lattice symmetry $\left({\mathbb{Z}}^{2},+\right)$ , with the difference being that lattice vectors are of the form A(t)z with $z\in {\mathbb{Z}}^{2}$ as time t progresses from the initial to final state. The matrix A(t) describes the evolution of lattice basis directions during deployment. Whereas a trellis has one degree of freedom and the edge lengths remain constant much like a scissor jack, this is not a requirement for other deployable lattices.

In the case of a scissor jack or deployable lattice, discrete translational symmetry groups describe the structure at a specific state of deployment, but continuous parameters such as lattice angles and lengths describe the state of evolution of the structure. It is not an either-or situation in regards to discrete rotational or translational symmetry. Crystallographic, rod and layer symmetry groups describing various states of matter consist of both discrete roto-reflections and translations. It is well known in the field of macromolecular crystallography that a search over relevant continuous rotations and translations can be used to construct phasing models to complement X-ray diffraction data. In these molecular replacement computer searches, the size of the search space is reduced significantly by taking into account symmetry.

**Figure 3.**
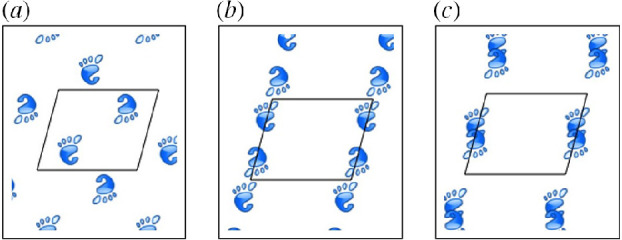
Three Instances of Γ=p2 wallpaper symmetry with footprint motif moved by different g∈G . (*a*) Motifs inside standard unit cell and not in collision, (*b*) motifs at edges of unit cell and not in collision and (*c*) motifs at edges of unit cell and in collision. Reprinted from Chirikjian GS, Shiffman B. 2014. Collision-free configuration-spaces in macromolecular crystals. *Workshop on Robotics Methods for Structural and Dynamic Modelling of Molecular Systems, Robotic Science and Systems*, Berkeley, CA, July 12, 2014.


Figure 3 shows different ways in which a footprint pattern can be laid down in different ways while preserving the same symmetry group. In all three subfigures, the same non-orthogonal unit cell is observed with a black frame, and the same wallpaper group Γ≅p2 describes the symmetry in each subfigure. The notation p2 describes the combination of integer translations in two independent directions (along the two edges of the unit cell) in combination with 180° rotations. Here, ‘ ≅ ’ is used to denote that Γ is of the same ‘type’ of symmetry group as the ‘standard’ p2 that has elements expressible as matrices of the form

$$\tau \left(i,j,k\right)=\left(\begin{array}{ccc} {\left(-1\right)}^{k} & 0 & i\\ 0 & {\left(-1\right)}^{k} & j\\ 0 & 0 & 1 \end{array}\right),$$

where i,j,k∈ℤ . The group operation is then just multiplication and closure under multiplication and inversion can be seen immediately because


$$\tau \left(i_{1},j_{1},k_{1}\right)\;\tau \left(i_{2},j_{2},k_{2}\right)=\tau \left(i_{1}+{\left(-1\right)}^{k_{1}}i_{2},j_{1}+{\left(-1\right)}^{k_{1}}j_{2},k_{1}+k_{2}\right).$$

This describes motion in an orthogonal lattice. To get a general non-orthogonal lattice while fixing the horizontal direction, then a specific Γ≅p2 is obtained by running through all elements τ(i,j,k) and producing


$$\gamma \left(i,j,k\right)=\alpha \;\tau \left(i,j,k\right)\;\alpha^{-1},$$

where


(1.1) $$\alpha =\left(\begin{array}{ccc} a & b\cos\phi & 0\\ 0 & b\sin\phi & 0\\ 0 & 0 & 1 \end{array}\right).$$

If a,b,ϕ depend on time then the application of α to the lattice ${\mathbb{Z}}^{2}$ describes its evolution into a different (non-orthogonal) lattice. Γ denotes a specific instance of type p2 with fixed values of a,b,ϕ . The closure of Γ under multiplication and inversion of elements follows immediately from the form closure of τ given above.

The group Γ is discrete and countably infinite. In contrast, the continuous group of motions of the plane, or special Euclidean motions, G=SE(2) is a Lie group. This continuous group describes the differences between these subfigures. Elements of SE(2) can be described as matrices of the form


(1.2) $$g(x,y,\theta )=\left(\begin{array}{ccc} \cos\theta & -\sin\theta & x\\ \sin\theta & \cos\theta & y\\ 0 & 0 & 1 \end{array}\right).$$

If x,y,θ depend on time, then the result is a planar motion trajectory. The product of two such matrices $g(x_{1},y_{1},\theta_{1})$ and $g(x_{2},y_{2},\theta_{2})$ will produce another of the same form and likewise for matrix inversion. Note also that elements of Γ are special cases of the above. As Γ is a group in its own right and Γ⊂G , the notation Γ<G is used to distinguish between mere inclusion as a subset and Γ being a group in its own right. The statement ‘ Γ<G ’ is read as ‘ Γ is a subgroup of G .’

The application of different group elements g∈G to an individual footprint pattern followed by discrete repetition given by Γ means that a different ‘right coset’ Γg describes each of these subfigures. Such a coset is generated by applying every element of Γ to g on the left (or, equivalently, applying g to every element of Γ on the right). The full freedom of G is described by arbitrary translations and rotations in the plane, (x,y,θ) , but in all figures shown θ is held fixed (i.e. the footprint pattern changes position but not orientation). These figures, as well as those that follow, were generated using the ‘Eschermoble’ iphone app [7].

From figures 1 and 2, we see that deployment can be described by transitioning between different symmetry groups of the same type and from figure 3, we see that continuous freedom can exist even while preserving a specific discrete symmetry group. These two phenomena can be combined. That is, the footprint patterns could change their relative motions while the lattice parameters of the discrete symmetry group are allowed to change. For example, specific symmetry groups in the class p2 are defined by scaling the translations along the two edge directions, as well as changing the angle between them. Therefore, when considering the design of a planar deployable mechanism, freedom exists in terms of g consisting of planar rigid-body displacements given by (x,y,θ) , as well as the freedom to change lattice parameters (a,b,ϕ) .

**Figure 4. F4:**
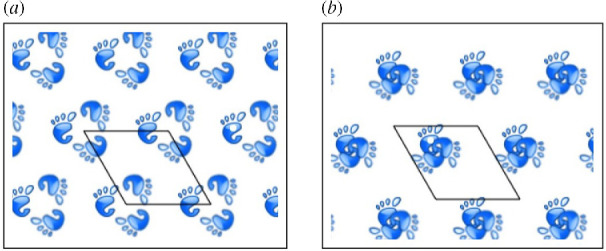
Two instances of Γ=p3 wallpaper symmetry with footprint motif moved by different g∈G . (*a*) Motifs not in collision and (*b*) motifs in threefold collision. Reprinted from Chirikjian and Shiffman, 2014. Collision-free configuration-spaces in macromolecular crystals. Workshop on robotics methods for structural and dynamic modelling of molecular systems, robotic science and systems, Berkeley, CA, July 12, 2014.

Figure 4 shows another example of this phenomenon with Γ in class p3 , consisting of two directions of integer translations and 120° rotational symmetry. Unlike p2 where the translational displacements in the two independent directions can be different, here they must be the same. Also, the angles between these directions (which define the lattice cell geometry) are fixed. The only freedom in defining this class of discrete symmetries is a single-scale factor. That said, if the scale is fixed, there is still freedom to move by g∈G . Some of these motions result in collision-free arrangements and others result in collision. Assessing the resulting ‘collision zones’ can be used as a tool in the design of deployable mechanisms and metamaterials as outlined later in this article. But first, more examples are provided to ground the mathematics that will follow.

**Figure 5.**
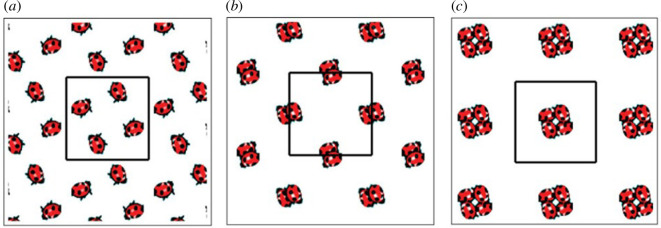
Three Instances of Γ=p4 wallpaper symmetry with ladybug motif moved by different g∈G : (*a*) no collision, (*b*) pairwise/twofold collision and (*c*) fourfold collision. Reprinted from Chirikjian and Shiffman [8].

Figure 5 shows three configurations, each with the same p4 symmetry. p4 consists of two orthogonal translations and rotations by increments of 90°. As with p3 , the only freedom in defining the shape of the lattice is a single-scale factor. Here, the motif is a ladybug. Unlike the p3 case, the modes of collision include twofold and fourfold. This is because four is divisible by two whereas three is prime.

**Figure 6.**
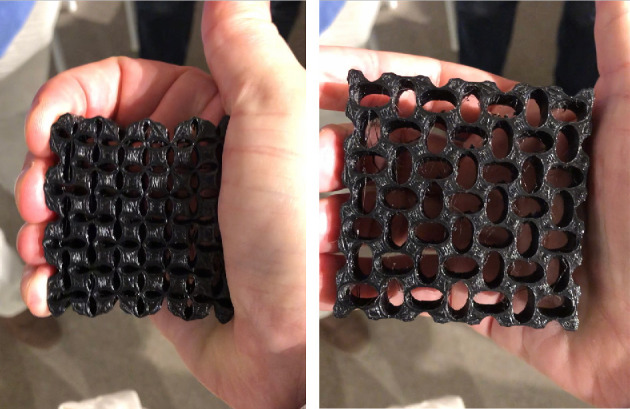
Preservation of wallpaper symmetry group type during deformation of an auxetic metamaterial (in the hand of the author on a visit to the Tachi Lab at University of Tokyo).

In contrast to figure 5 in which the scale of the lattice is held fixed and the size and shape of the motifs (the ladybugs) is constant, figure 6 shows the deformation of a metamaterial. As it deforms, the symmetry type is still p4 but the lattice size changes. Unlike in figure 5, here g∈G is constant, and the motifs (ellipsoidal holes) change shape during the deformation. One unit cell consists of a pair of holes with long axis in the vertical direction and a pair in the horizontal direction, with each member of a pair diagonal from each other.

Another example is the group p6 . Like p3 and p4 , this family only has one freedom to change its geometry, and that is through scaling. As 6 is divisible by 2 and by 3, the different kinds of collisions that can take place as g∈G is varied are twofold (like in p2 ) and threefold (like in p3 ).

### (b) Non-lattice deployable structures

Not all deployable structures consist of variable lattices or trellises consisting of translational repeats. Many deployable structures have discrete rotational symmetries at each stage of deployment and preserve the position of a point and axes of rotation passing through that point while deploying. An everyday example of this is an umbrella where the axis of rotational symmetry coincides with the handle, and the preserved point is the distal vertex. In the design of an umbrella, only one sector of a circular disk needs to be considered and then repeated by rotational symmetry. In an umbrella (without a symmetry-breaking handle), the discrete rotational symmetries about one fixed axis explain how to rotate to achieve an identical appearance at a specific state of deployment, but will not explain the evolution of the deployment itself, which involves the intricacies of mechanical linkage design.

**Figure 7.**
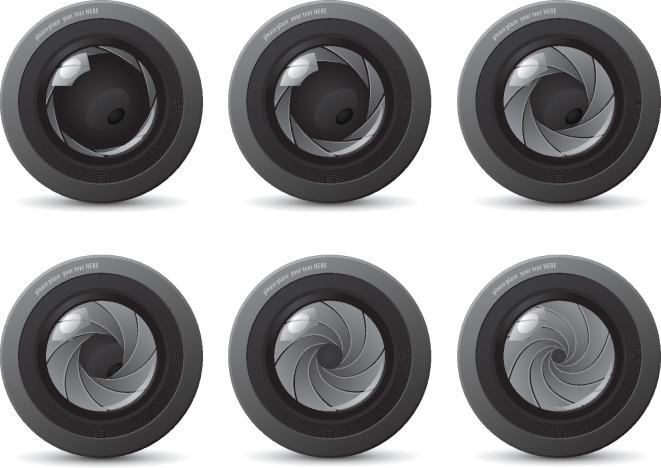
The aperture of a mechanical camera Iris (*source*: shutterstock.com purchased with license).

A related deployable mechanism with rotational symmetry is the iris of a camera, as shown in figure 7. Multiple designs exist. Some involve gears and others involve pins constrained to move in slots. As mesmerizing as a mechanical iris can be, it is in many ways simpler than an umbrella. The mathematical analysis of a mechanical iris is similar to that of a kaleidoscope or the symmetry-preserving motions in figures 3 [Fig F4]–5 and 8. The coordination of motions to preserve symmetry in this case has a fixed rotational symmetry group, Γ<SO(2) . Not all systems with rotational symmetry are planar with a single rotation axis. More complex examples are described below.

**Figure 8.**
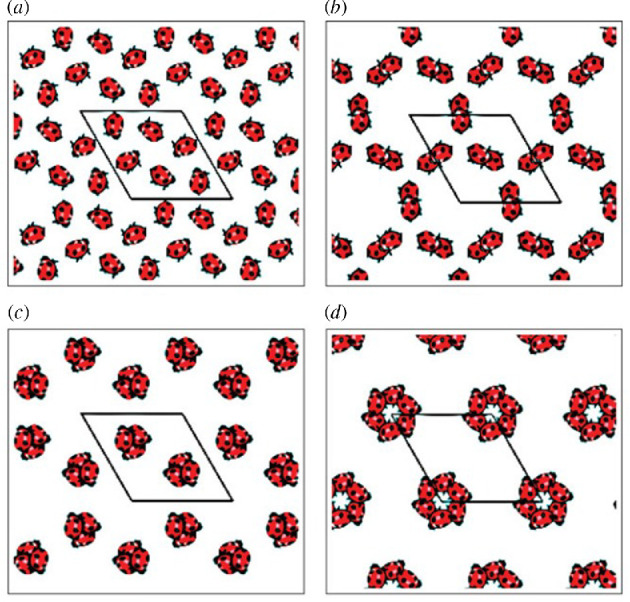
Four instances of Γ=p6 wallpaper symmetry with ladybug motif moved by different g∈G . Reprinted from Chirikjian, G.S. and Shiffman, B., Chirikjian and Shiffman [8].

**Figure 9.**
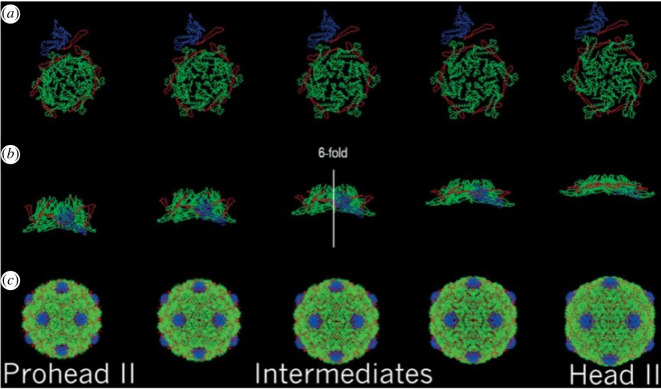
Maturation of the HK97 bacteriophage capsid. Reprinted from Kim *et al*. [5].

The finite group of rotational symmetries of the icosahedron describes how to rotate a static icosahedron back into itself. While this symmetry alone does not describe how an icosahedral virus expands or the relative locations of, or deformations of, the capsomere proteins during that expansion, it does allow for the simplification in analysis during maturation to divide the capsid into the most fundamental subunits (the so-called asymmetric units) as shown in figure 9. This allows for the division of the capsid into 60 identical pieces that move in concert to preserve symmetry. In a sense, nature discovered icosahedral symmetry eons before the Pythagoreans did.

**Figure 10.**
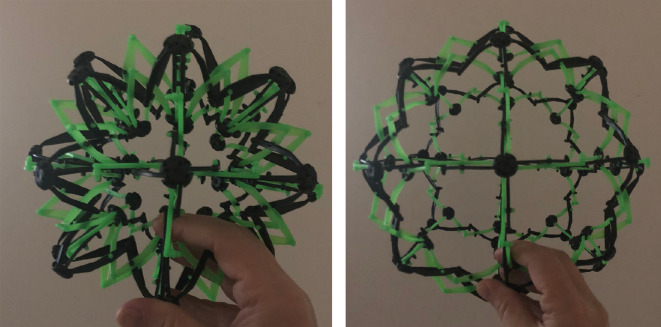
Cubo-octahedral symmetry of expanding Hoberman sphere.

The same idea applies to Hoberman spheres. The one shown in figure 10 (purchased and held by the author) has cubo-octahedral rotational symmetry. Therefore, the design and simulation during deployment reduces to the study of 1/24th of the total. This division is very different to how the Hoberman sphere was devised, which involved first the construction of expanding circular hoops that could be interlocked as great arcs on a sphere.

Other deployable structures have different kinds of non-lattice symmetries. These include deployable helical antennae, which have continuous screw symmetries at each stage of deployment, the pitch of which changes during deployment.

### (c) Literature review

This article establishes relationships among a number of fields and develops mathematical tools relevant to them all. The related literature is, therefore, immense and highlights from each related field are given below.

#### (i) Deployable mechanisms

An essential property of deployable mechanisms is that they can transform from a relatively compact state to one that has a much greater area or volume. They can consist of discrete mechanical linkages or can have a more continuous structure such as deployable helical antennae [9–31]. A closely related area is that of tensegrity structures [32,33]. Sometimes deployable mechanisms are used as components in ‘lattice type’ modular self-reconfigurable systems [34–37].

#### (ii) Origami mechanisms

Origami (paper folding) is an ancient Asian art form. In the past decade or so this has inspired a number of engineering researchers to use principles of origami to design structures that are light weight and transformable. Whereas traditional origami is based on thin paper, more recent engineering origami includes thick components [12,38–48].

#### (iii) Metamaterials

The relatively new topic of metamaterials has become very popular in recent years. Whereas the properties of traditional engineering materials are defined by their bulk materials, metamaterials are structured at length scales larger than the classical grain size to include voids, flexures, honeycomb structures, etc., in order for the larger structures made from metamaterials to have different properties than the underlying bulk materials. Applications include acoustical properties such as greater sound absorption, damping of electromagnetic radiation and counter-intuitive material properties such as negative Poisson’s ratio. The fabrication of metamaterials via three-dimensional printing and other methods is also an area of active interest. For literature on these topics see [19,49–57].

#### (iv) Crystallography

The theory of crystal symmetry is very well developed and fully documented in the International Tables [58]. It relies heavily on fundamental group theory, but has a richness in its own right, which originates from the intricacies of the geometry of crystals. In crystallography, a motif is repeated regularly with combinations of discrete translational, rotational and mirror symmetries. Crystallography can be treated from different perspectives ranging from more algebraic to more geometric. A full spectrum of the literature is described in [58–68]. The interplay between space groups and the groups of continuous motions plays a role in macromolecular crystallography as described in [8,69–77]. The mathematics at the core of the present formulation builds on the ideas presented in those papers.

#### (v) Low dimensional geometry and topology

A class of mathematical objects of extensive study in mathematics for the past 150 years is that of a manifold. A manifold is a generalization of a surface which locally resembles Euclidean space at every point. Many manifolds arise in one of two ways: (i) as subsets within higher dimensional Euclidean spaces (e.g. a sphere as a set of points equidistant from the origin) or (ii) as quotients of simpler spaces without regard to embedding in an Euclidean space. As an example of the latter, the manifold corresponding to the set of 3×3 rotation (a.k.a. special orthogonal) matrices, SO(3) , can be characterized by starting with the unit sphere in four-dimensional Euclidean space (the quaternion sphere) and ‘glueing’ antipodal points. Alternatively, the manifold for SO(3) can be identified as the solid ball of radius π in three-dimensional space and glueing antipodal points on the surface. Either way, the resulting manifold no longer lives in the Euclidean space that the original sphere or ball did.

This intuitive way of treating manifolds has been advocated by great mathematicians including Poincaré and Thurston, among others, as described in [78–86].

The glueing process can be made rigorous as a quotient procedure with an appropriate group action. For example, the glueing of antipodal points on the quaternion sphere corresponds to identifying the unit vector u with −u . The set {−1,1} together with the operation of scalar multiplication is abstractly the same group as the set {0,1} with operation of addition modulo 2, which is denoted ${\mathbb{Z}}_{2}$ . Therefore, mathematicians write that $SO(3)≅S^{3}/{\mathbb{Z}}_{2}$ .

More generally, a sphere can be divided by a more complicated finite group such as the group of symmetries of the tetrahedron, cube or icosahedron. The resulting manifolds are called spherical space forms [86]. Or, if an Euclidean space is quotiented by special kinds of crystallographic groups called Bieberbach groups [78], the resulting manifolds are called Euclidean space forms.

When quotient Euclidean space by non-Bieberbach crystallographic groups, the result is no longer a manifold, but is instead a generalization called an *orbifold* [84].

### (d) Overview of subsequent sections

A mathematical formulation of transitions/deployments is developed in this article. In the remainder of this article, formal definitions of group theory are reviewed, and used as a language to describe the sorts of deployments and deformations observed earlier in this section.

It is shown that if X is a continuous space such as Euclidean space or a sphere, G is a continuous group of motions acting on X (such as the space group of Euclidean motions or pure rotations), and Γ is the type of the discrete subgroup of G describing the symmetries of the deploying structure, then the evolving structure can be described by a time-dependent subgroup of G computed as $\Gamma_{\alpha_{t}}:=\alpha_{t}\Gamma \alpha_{t}^{-1}<G$ , where $\alpha_{t}$ is an affine transformation that varies with time, t . Then, instead of considering the whole deployable structure in X , a ‘sector’ of it that lives in the orbit space $\Gamma_{\alpha_{t}}\backslash X$ can be considered. This orbit space can be considered as not only the smallest part of the structure that can be used to reproduce the whole (the so-called asymmetric unit in crystallography jargon), but also implicitly defines a rule to ‘glue’ the pieces together. For example, instead of considering a whole umbrella or scissor jack, it is sufficient to consider only one subunit, which is then repeated by the action of the discrete symmetry group. And when considering motions, instead of taking all motions in G , only representatives from ‘right cosets’ in the much smaller ‘right coset space’ $\Gamma_{\alpha_{t}}\backslash G$ need to be considered. The language used here, which may be familiar to mathematical crystallographers, pure mathematicians working in geometry and topology, theoretical physicists and some solid-state physicists, may be unfamiliar to more applied researchers. However, the intention behind this article is to develop these tools and to make them accessible to assist practitioners in both the design and simulation of time-evolving structures that preserve symmetry types.

A short summary of the main results is now presented with an eye towards comprehensibility prior to introducing rigour, and the subsequent sections aim to formalize these concepts and provide sufficient detail to use them in examples. This is followed by background sections that can be skipped by readers familiar with group theory.

The remainder of this article is structured as follows. Section 2 introduces general notation from group theory. Section 3 presents a brief review of discrete groups of motions describing crystallographic symmetry.

## 2. Groups, actions and quotient spaces

The concept of a group is fundamental in mathematics. A group consists of a set, G , and an operation, ∘ , that can combine any two elements of G to produce an element of G . That is, given arbitrary elements $g_{1},g_{2}\in G$ , then $g_{1}\circ g_{2}\in G$ . In addition to this closure property, this combination of the set G and operation ∘ must satisfy three additional properties:

(i) the set must contain a special ‘identity element’, e , such that for every g∈G we have


e∘g=g∘e=g;

(ii) for each g∈G , there exists a unique element $g^{-1}\in G$ such that


$$g^{-1}\circ g=g\circ g^{-1}=e;$$

(iii) for arbitrary $g_{1},g_{2},g_{3}\in G$ , the associative property


$$g_{1}\circ \left(g_{2}\circ g_{3}\right)=\left(g_{1}\circ g_{2}\right)\circ g_{3}$$

holds.

When all of these properties hold, the combination of set and operation, (G,∘) is called a group. If property 2 is not satisfied, it is called a semigroup, or if property 3 is not satisfied it is called a quasigroup.

In this article, all of the sets G consist of invertible square matrices and the operation is matrix multiplication and instead of writing $g_{1}{}^{\circ}g_{2}$ we write $g_{1}g_{2}$ . With the identity matrix serving as identity element, all of the group properties hold as long as closure is satisfied. In cases such as this where the group operation is understood, we write G as both the group and the set rather than the more cumbersome (G,∘) .

Within a group G there are special subsets that are themselves smaller groups called subgroups. This was demonstrated in the introduction where the group consisting of elements of the form of g has as a subgroup those elements of the form τ . If H is a subgroup of G then we write H<G or G>H .

### (a) Cosets and double cosets

Given H<G , then G can be partitioned into left or right cosets. For any given g∈G , the left coset containing G is defined as


gH≐{gh | h∈H}

and a right coset is defined as


Hg≐{hg | h∈H} .

That is, left and right cosets are generated by multiplying g on the left or right of every element of H . In this article right cosets will have specific applicability to the problem of deployable structures and results will be stated in terms of right cosets, though in abstract group theory, they apply equally well to left cosets.

In general gH≠Hg , but equality can hold in special cases. A special kind of subgroup for which each left coset is equal to the corresponding right coset is called a normal subgoup, and denoted as N⊲G .

It is possible for two different elements $g_{1},g_{2}\in G$ to generate the same coset, e.g. $g_{1}H=g_{2}H$ . If all distinct cosets are collected, the result is called a coset space. The left coset space is


G/H={gH | g∈G}

and the right coset space is


H∖G={Hg | g∈G}.

If H and G are finite groups, then number of cosets in a coset space, |H\G|=|G/H| is equal to the ratio |G|/|H| . This is Lagrange’s famous theorem.

A fundamental elementary result of group theory is that each right coset is distinct. That is, either $Hg_{1}=Hg_{2}$ or else $Hg_{1}\cap Hg_{2}=\emptyset$ . A consequence of this is that G can be divided into right cosets. Then the group can be reconstructed as


(2.1) $$G=\underset{Hg\in H\backslash G}{\cup }Hg\;.$$

When G is a Lie group and H is a non-trivial discrete subgroup (containing more than just e ), the resulting coset space will have the same dimension as G but smaller volume. If H is a Lie subgroup then the coset space will have dimension that is the difference in the dimension of G and H .

In the special case, when N⊲G then G/N=N\G and the resulting coset space is itself a group with group operation on the cosets defined as $(g_{1}N)(g_{2}N)=(g_{1}g_{2})N$ . The resulting group is called the quotient group, and is denoted as $\frac{G}{N}$ .

For example, when G=p4 the subgroup consisting of translations that move the square lattice back into itself is a normal subgroup and the quotient group $\frac{p4}{{\mathbb{Z}}^{2}}$ is abstractly the same group as the cyclic group $C_{4}$ consisting of 90° rotations. And, when G=SE(2) , the subgroup consisting of all planar translations is a normal subgroup and the quotient group $\frac{\text{SE}\left(2\right)}{R^{2}}$ is abstractly the same as the planar rotation group SO(2) .

### (b) Discrete subgroups of Lie groups and the concept of fundamental domains

The partitioning of G into cosets and the reconstruction of G from cosets as per (iii) is a standard concept in group theory. Less standard, though known, is that when G is a continuous (Lie) group and Γ<G is a discrete subgroup, there is an alternative way to partition G .

Given a rule σ:Γg→G for picking one element of Γg of the form s=σ(Γg)∈G , all of these selected elements can be collected into a subset of G . When the selection rule is defined such that this subset is a continuous region in G , then this subset is called a *fundamental domain* and is denoted as $F_{\Gamma \backslash G}\subset G$ . This can be thought of as a block of sorts inside of G . When the boundaries of the block are ‘glued’ together in an appropriate way, the result is the same manifold defined by Γ\G .

Then, the whole group G can be reconstructed from shifted copies of these fundamental domains as


(2.2) $$G=\underset{\gamma \in \Gamma }{\cup }\gamma \cdot F_{\Gamma \backslash G}\;.$$

Concrete ways for constructing fundamental domains when G is a group of rigid-body displacements of Euclidean space and Γ is a crystallographic space group have been studied extensively in [8,69–77].

In all of the footprint and ladybug figures shown earlier, G=SE(2) , the motions observed can all be taken in $F_{\Gamma \backslash G}$ , where Γ is one of p2,p3,p4,p6 . There is no need to consider motions outside of this fundamental domain. Although the product of two elements of $F_{\Gamma \backslash G}$ is not necessarily in $F_{\Gamma \backslash G}$ , it is always possible to bring the result back inside by multiplying with an appropriate γ∈Γ on the left.

### (c) Asymmetric units and orbifolds

As seen in the previous section, a discrete subgroup can divide a continuous group into fundamental domains. Similarly, a discrete group can divide a space other than a parent group into disjoint domains. This requires the concept of a *group action*. Given a group G , an action of this group on a set X is an operation that produces a new element of X and also satisfies an additional property. Namely, if g∈G and x∈X , then g⋅x∈X and


$$g_{1}\cdot \left(g_{2}\cdot x\right)=\left(g_{1}\circ g_{2}\right)\cdot x\;.$$

This associativity-like condition is a kind of compatibility between the group G and the set X .

If G is a continuous group and Γ<G is a discrete subgroup, then Γ inherits the ability to act on a set X . Then, Γ can divide X into regions (fundamental domains) in a similar way that it could divide G . This concept is well known in crystallography where space-group symmetries divide Euclidean space, X , into small non-overlapping blocks called *asymmetric units*. These are not unique; there are an infinite number of ways to construct these tiles, denoted here as $F_{\Gamma \backslash X}$ . One way is to apply Γ to the origin 0∈X , and to construct Voronoi regions around the resulting constellation of points.

Then, Euclidean space can be reconstructed by the union of all of these blocks as


$$X=\underset{\gamma \in \Gamma }{\cup }\gamma \cdot F_{\Gamma \setminus X}.$$

If the boundaries of $F_{\Gamma \backslash X}$ are glued together, the resulting mathematical object Γ\X is called an orbifold [79,84,85]. If Γ acts on X leaving no points fixed (i.e. for all γ≠e implies γ⋅x≠x for all x∈X ) then Γ\X will be a manifold.

### (d) The group of handedness-preserving Euclidean motions, SE(n)

The group of rigid-body motions in n -dimensional Euclidean space, G=SE(n) , consists of rotation–translation pairs of the form g=(R,t) where R∈SO(n) and $t\in {\mathbb{R}}^{n}$ . Such motions can be represented using (n+1)×(n+1) homogeneous transformation matrices of the form


$$H(g)=\left(\begin{array}{cc} R & t\\ 0^{T} & 1 \end{array}\right).$$

These matrices encode the group law as matrix multiplication:


$$H\left(g_{1}\circ g_{2}\right)=H\left(g_{1}\right)H\left(g_{2}\right).$$

The action of g∈SE(3) on a position $x\in {\mathbb{R}}^{n}$ is


g⋅x=Rx+t.

Similarly, if B is a solid body, then g⋅B denote the action of g on the totality of positions contained in B .

### (e) Group-theoretic methods in the analysis of mechanical linkages

Group-theoretic methods have been employed in mechanism and machine theory in a variety of ways in the past. In particular, symmetry properties of linkages have been studied in [87–89]. Group theory in this context usually relates to the symmetry properties of individual joints that comprise a linkage and the impact of those symmetries on the mobility of the overall linkage. These analyses relate to low-dimensional Lie subgroups of the group of rigid-body displacements describing the mobility of lower kinematic pairs. Interestingly, the work of Schoenflies (e.g. [90,91]) from more than 100 years ago has impacted both kinematics and crystallography. These fields have largely evolved along divergent paths since then, with the kinematics community studying continuous motions and the crystallography community studying discrete symmetries. In some rare instances, in which researchers in the kinematics community have studied periodic linkages, some ideas from crystallography such as the theory of characters of crystallographic point groups have been adapted [92,93]. Characters can be computed as the trace of a group representation matrix. The full theory of crystallographic space group representations is known [94], as is the representation theory of the group of rigid-body motions [95]. The present work takes a different approach when reducing symmetries in deployable structures and metamaterials. This work blends ideas from the study of continuous and discrete symmetries using the concept of quotient spaces of Euclidean groups by crystallographic groups rather than the theory of group characters or representations.

## 3. Crystallographic symmetry

At first glance, n -dimensional Euclidean space $X=E_{n}$ appears to be a bland and structureless blank slate. But hiding within this continuum is a significant structure that emerges when an origin and coordinate system is introduced and used together with a ruler for measuring distances. For example, the vector space structure of ${\mathbb{R}}^{n}$ enables us to more easily realize that the underlying space $E_{n}$ is closed under the action of Euclidean isometries of the form


x→Qx+t

where Q is an orthogonal matrix ( $QQ^{T}=I_{n}$ , the n -dimensional identity matrix) and t is a translation vector. (Unlike R in SE(n) , here Q can be a roto-reflection with detQ=−1 .) The set of all such Q matrices is the orthogonal group O(n) , which is closed under the operation of matrix multiplication.

That is, $Q_{1},Q_{2}\in O(n)\to Q_{1}Q_{2},Q_{1}^{-1}=Q_{1}^{T}\in O(n)$ . Rotation matrices are those orthogonal matrices that have the additional constraint that detQ=+1 , and the set of all such n -dimensional rotations is denoted as SO(n) .

The group of Euclidean isometries, G=E(n) , has elements of the form g=(Q,t) , and has group law


$$g_{1}\circ g_{2}≐\left(Q_{1}Q_{2}\;,\;Q_{1}t_{2}+t_{1}\right).$$

It is easy to see that SE(n)<E(n) . Both of these groups are semi-direct products:


$$E\left(n\right)=R^{n}⋊O\left(n\right)\;\;\;\text{and}\;\;\;\text{SE}\left(n\right)=R^{n}⋊SO\left(n\right)\;.$$

Effectively in the present context, this means two things: (i) the subgroup of pure translations is normal, ${\mathbb{R}}^{n}⊲E(n)$ and $R^{n}⊲\text{SE}\left(n\right)$ , allowing us to write^1^


$$\frac{E\left(n\right)}{R^{n}}≅O\left(n\right)\;and\;\frac{SE\left(n\right)}{R^{n}}≅SO\left(n\right)$$

and (ii) every general pair can be written as a product of pure translations and rotoreflections as


(Q,t)=(I,t)∘(Q,0),

where I is the identity matrix.

Even more structure is endowed on the continuum by considering crystallographic lattices in analogy with how integers embedded in the real number line give rise to complicated objects such as the prime numbers. Discrete groups of isometries such as the 17 classes of planar wallpaper groups describe geometric tilings that repeat periodically under translation. Many of these tilings were constructed intuitively centuries ago in the Nasrid art of Alhambra. The three-dimensional versions of these are the 230 classes of crystallographic space groups. Of these 230, only 65 are subgroups of SE(3) . These handedness-preserving space groups are called Sohncke groups. A crystal (or wallpaper pattern) has more structure than only the underlying translational lattice. It also contains a ‘motif’ that occupies the void between lattice points. Such a motif might be Escher’s artwork or a protein in the context of a macromolecular crystal. The footprint and ladybug patterns in the figures of §1 of this article are motifs as well. A space group describes the discrete Euclidean isometries that carry one copy of a motif to another in a crystal.

Crystallographic space groups have intricate algebraic structures beyond the structure of E(n) . Denote a space group as Γ<E(n) . Its elements will have the form


$$\gamma =\left(Q_{\gamma }\;,\;t_{\gamma }+v\left(Q_{\gamma }\right)\right),$$

where


$$t_{\gamma }\in A{\mathbb{Z}}^{n}$$

is a lattice translation (with lattice shape defined by the invertible matrix A ) and $v(Q_{\gamma })$ is a translation by a fractional amount within one cell of the lattice.

The condition that Γ is closed requires that


$$v\left(Q_{1}Q_{2}\right)-R_{1}v\left(Q_{2}\right)-v\left(Q_{1}\right)\in L,$$

where $L=A{\mathbb{Z}}^{n}$ is the lattice. Another way to write this is


$$v\left(Q_{1}Q_{2}\right)≅R_{1}v\left(Q_{2}\right)+v\left(Q_{1}\right)\;\;\text{mod}\;L.$$

The group of translations of the lattice L is denoted as T . The group of rotoreflections consisting of elements $\left\{Q_{i}\right\}$ can be called the concrete point group, P , which is isomorphic to the abstract point group $\frac{\Gamma }{T}$ .

Interestingly, of the 230 classes of three-dimensional crystallographic space groups, most (157) are non-symmorphic, meaning that Γ≠T⋊P . The 73 which can be written as a semidirect product are called symmorphic.

Another special kind of space group is called a Bieberbach group, denoted here as $\Gamma_{B}$ . These groups have no fixed points. In other words, they have no pure rotoreflections of the form $(Q_{i},0$ . Except for the identity element, no element of a Bieberbach group applied to a point in $X={\mathbb{R}}^{n}$ will leave it fixed. Consequently, the space of orbits $\Gamma_{B}\backslash X$ is always a manifold. The group T is one of the Bieberbach groups but it is not the only one. (The quotient T\X is the torus.) Twelve others among the 230 classes of three-dimensional crystallographic space groups are Bieberbach. The elements of these Bieberbach groups include translations, screw displacements and glides (combinations of translation and reflection). Eight of these 12 are Sohncke groups also. Interestingly, almost half of all protein crystals have Sohncke Bieberbach groups as their symmetry.

Many intricate relationships exist between space groups and their subgroups. Of the 65 Sohncke groups, most (but not all) can be written as a semidirect product of a Bieberbach group and a subgroup of the concrete point group of the form [60]


$$\Gamma =\Gamma_{B}⋊S\;.$$

This has implications for how the space Γ\G can be described.

### (a) Motion spaces in molecular replacement

Lie groups describe motions (or ‘actions’ more generally), and can be viewed as objects that morph points in a space on which they act. The basic tenet of this article is that when the objects being morphed (such as a deployable structure) have discrete symmetry types that are preserved during the morphing, then the analysis can be simplified in two ways: (i) only a restricted set of motions acting on the structure needs to be considered; and (ii) only a reduced part of the structure needs to be considered. Group theory provides the terminology to formalize these intuitive concepts.

Given a Lie group, G , such as E(n) , O(n) , etc., and a discrete subgroup, Γ , such as a crystallographic space group or the group of roto-reflective symmetries of a Platonic solid, left and right cosets are defined for each g∈G as


gΓ≐{g∘γ | γ∈Γ}   and   Γg≐{γ∘g | γ∈Γ},

respectively. Each such coset is a subset of G . The set of all such cosets is called a coset space, quotient space or orbit space.

The left and right coset spaces are denoted as


G/Γ≐{gΓ | g∈G}   and   Γ∖G≐{gΓ | g∈G}.

Multiple values of g can produce the same coset. That is, it is possible that $g_{1}\neq g_{2}$ , yet $g_{1}\Gamma =g_{2}\Gamma$ . But if $g_{1}\Gamma \neq g_{2}\Gamma$ and if $g\in g_{1}\Gamma$ , then it must be that $g\notin g_{2}\Gamma$ . That is, G can be partitioned into cosets, each of which has the same size. Choosing one representative element per coset, it is possible to construct a continuous domain within G , called a fundamental domain denoted as $F_{\Gamma \backslash G}$ . Then


(3.1) $$G=\underset{g\in F_{\Gamma \setminus G}}{\cup }\Gamma g=\underset{\Gamma g\in \Gamma \setminus G}{\cup }F_{\Gamma \setminus G}.$$

and similarly for the left case.

If gΓ=Γg for every g∈G , then Γ is called a normal subgroup and then G/Γ=Γ\G is itself a Lie group. For example, if G=O(3) and Γ={−I,I} , then SO(3)=O(3)/Γ . Or if $G={\mathbb{R}}^{n}$ with vector addition as the group operation and $\Gamma ={\mathbb{Z}}^{n}$ , then ${\mathbb{R}}^{n}/{\mathbb{Z}}^{n}$ is the n -torus with the operation circular addition.

But such cases are rare. Usually Γ is not a normal subgroup of G , and the resulting coset spaces have no group operation. Nevertheless when G is a Lie group and Γ is a discrete subgroup, the coset spaces G/Γ and Γ\G are always manifolds.

Given two discrete subgroups of G , denoted as Γ and Δ , if Γ∩Δ={e} , the set containing the identity element of G , then the double-coset space


Γ∖G/Δ={ΓgΔ g∈G}.

is also a manifold where each double coset is defined as


ΓgΔ={γ∘g∘δ | γ∈Γ,δ∈Δ} .

Fundamental domains for double coset spaces can be constructed, and extensions of (3.1) can be written using the fact that


$$\underset{\delta \in \Delta }{\cup }F_{\Gamma \backslash G/\Delta }\delta =F_{\Gamma \backslash G}\;.$$

If G acts on a space, X , then it is also possible to divide that space into orbits. Formally, a group action ⋅ must satisfy $g_{1}\cdot \left(g_{2}\cdot x\right)=\left(g_{1}\circ g_{2}\right)\cdot x$ for every $g_{1},g_{2}\in G$ and x∈X . For example, when G=E(n) and $X={\mathbb{R}}^{n}$ , the group action is g⋅x=Qx+t . Orbit spaces of discrete groups can be defined as Γ\X={Γx |,x∈X} with Γx defined similarly as Γg , but with group action replacing group operation, and the corresponding fundamental domains $F_{\Gamma \backslash X}$ can be defined as well. These can be thought of as tiles, such as the asymmetric units of space groups in Euclidean space, or the triangular subdivisions of the sphere under the action of symmetry groups of the Platonic solids. The orbit spaces Γ\X generally will not be manifolds, and are called orbifolds. In the special case when the action of Γ on X is ‘torsion free’, then the result will be a manifold. This is because in this case $\gamma_{1}x=\gamma_{2}x$ implies $\gamma_{1}=\gamma_{2}$ .

When Γ is a ‘Bieberbach’ group (a special kind of crystallographic space group without pure rotations or reflections) and $X=E_{n}$ then the resulting quotient space will be a flat manifold called an Euclidean space form. Or if Γ is a group of rotational symmetries of a Platonic solid and X is the sphere, then the quotient space will be a spherical space form.

Whereas a deployable structure may live in the space X , a section of it that is reproduced under symmetry lives in $F_{\Gamma \backslash X}$ . Whereas G may describe all possible motions, the relevant motions can be restricted to $F_{\Gamma \backslash G}$ .

It has been shown in recent work that one can choose $F_{\Gamma \backslash G}$ in many different ways such as


$$F_{\Gamma \setminus G}=F_{T\setminus X}\times F_{P\setminus SO\left(3\right)},$$

(i.e. taking translations in the unit cell and rotations in a sector of SO(3) with 1/|P| the volume) or


$$F_{\Gamma \setminus G}=F_{\Gamma \setminus X}\times SO\left(3\right),$$

(i.e. considering only translations in the crystallographic asymmetric unit and spanning all rotations), or if $\Gamma =\Gamma_{B}⋊S$ then


$$F_{\Gamma \backslash G}=F_{\Gamma_{B}\backslash X}\times F_{S\backslash SO(3)}\;.$$

Other choices exist as well.

The fundamental domains described above are the search spaces in the computational macromolecular crystallographic phasing method called molecular replacement. These particular realizations were introduced in [8,69–77]. Coset spaces of the form Γ\G also played a role in the formulation of Hilbert’s 18th problem, as well as in Louis Michel’s theory of liquid crystals [96]. In this article, they play the role of how to arrange the links in deployable structures or voids in metamaterials such that they do not collide during shape change.

### (b) Deployable lattice structures

As a lattice structure deploys, one of three possibilities exist: (i) Either the lattice itself is held fixed and the mechanical components, or bodies, B , rearrange (as in the footprint and ladybug figures shown earlier); (ii) the lattice changes and the relative motions within stay constant; (iii) both the lattice changes shape/size and the relative motion of components changes as well. This is described simply in a single expression


$$\left(\Gamma g\right)B\to \left(\Gamma^{\prime }g^{\prime }\right)B^{\prime }.$$

Here, $\Gamma^{\prime }$ can either be the constant Γ or it can be dynamic and changes as $\Gamma^{\prime }=\Gamma_{\alpha (t)}$ . Similarly, $g^{\prime }=g(t)$ can be dynamic. Even the constituent bodies can change shape with time as B=B(t) . An example of this is the ellipsoidal voids in the metamaterial shown in figure 6. It can also be that the body is a linkage consisting of multiple rigid links that move relative to each other owing to the rotation of joints or roll against each other as cams do. Then, the bodies can change shape over time. In any case, there are two constraints when considering coordinated symmetrical deployment: (i) the bodies cannot interpenetrate with the interiors of symmetry mates; ii) the surfaces of bodies must remain in contact with the surfaces of their symmetry mates so as not to form isolated islands to ensure that the whole structure remains connected during deployment. This motivates the theory of kissing constraints developed in the next section.

## 4. General theory of symmetrically arranged kissing bodies

As established earlier, the problem of designing a symmetry-preserving deployable mechanism can be reduced to that of studying one sector or asymmetric unit. A mechanism or linkage is composed of multiple individual bodies or links. As the mechanism successfully deploys, these bodies must not collide. It is sufficient to ensure this by examining what happens in a single asymmetric unit. Linkages can be constructed in many ways including by pinning links to form fixed joints or allowing rolling with gears or cams. Umbrellas typically involve the former, whereas a camera iris typically involves the latter.

In this section, a general theory for handling the case of rolling is addressed. This case is chosen for two reasons: (i) The construction of symmetrical linkages with fixed joints is less challenging because it reduces to the classical design of linkages, and symmetries are imposed rather easily. The construction of symmetrical linkages with fixed joints is less challenging, as it reduces to the classical design of linkages and the symmetries imposed rather easily; (ii) the case of kissing constraints applies equally well to symmetrical deployable linkages composed of solid bodies, as well as metamaterials in which the roll of the bodies is replaced with voids. In that case, the voids may have shape and size that can vary with time. Hence, in the discussion below, the bodies need not be thought of as objects that have fixed shape and size.

Given two closed convex bodies, $B_{1}$ and $B_{2}$ , each described in a world reference frame, the subset of SE(n) that will result by holding $B_{1}$ fixed and moving $B_{2}$ such that only the boundaries of the bodies intersect is denoted as $K_{1,2}\subset \text{SE}\left(n\right)$ , and is referred to here as the ‘kissing space’. That is,


$$K_{1,2}≐\left\{k\in \text{SE}\left(n\right)\;|\;\partial B_{1}\cap k\cdot \partial B_{2}\neq \emptyset \;\;\;\text{and}\;\;\;B_{1}^{\circ }\cap k\cdot B_{2}^{\circ }=\emptyset \right\},$$

where $B_{i}^{{}^{\circ}}$ is the largest open subset of $B_{i}$ , i.e. $B_{i}^{{}^{\circ}}=B_{i}-\partial B_{i}$ (the set theoretic difference of the closed body and its boundary). From this it follows that


$$K_{2,1}=K_{1,2}^{-1},$$

and if the bodies are identical, there is no need for subscripts and


$$K=K^{-1}.$$

**Figure 11.**
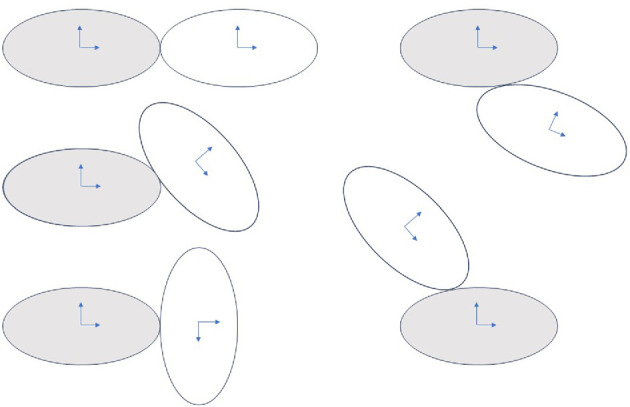
The kissing space of two identical ellipsoids with motions shown relative to the shaded one.

The kissing space for the case of two identical ellipsoids is shown in figure 11. Here, the shaded one is held fixed and the other moves relative to it while maintaining point contact. The kissing space has one dimension lower than the group of motions. In two dimensions the kissing space is two-dimensional. In three dimensions, the kissing space is five-dimensional.

If $B_{1}$ and $B_{2}$ are convex and $B_{1}$ is fixed at the identity frame and $B_{2}$ is moved by $k=(R_{k},t_{k})$ , then the kissing space is explicitly described as [8,97]


(4.1) $$K=\{R_{k}\in SO(3),t_{k}\in B_{1}+(-R_{k}\cdot B_{2})\}\;.$$

That is, $R_{k}$ is an arbitrary rotation and $t_{k}$ must lie in the Minkowski sum of $B_{1}$ and a rotated and centrally inverted version of $B_{2}$ .

When considering identical copies of convex bodies that are in contact with each other, and are also arranged with crystallographic symmetry, as described by the space group Γ<SE(n) , it can be shown that the motion g∈SE(n) which puts a body B in contact with its symmetry mate (γ∘g)⋅B must satisfy the constraint equation


(4.2) $$g^{-1}\circ \gamma \circ g\;\in \;K.$$

When there are multiple such contacts, we write


(4.3) $$g^{-1}\circ \gamma_{i}\circ g=k_{i}\;\in \;K.$$

Here, $\gamma_{i}$ and $\gamma_{j}$ for i≠j are only written if they are independent in the sense of not being conjugates of each other or powers of each other, because those can be obtained from each other.

**Figure 12.**
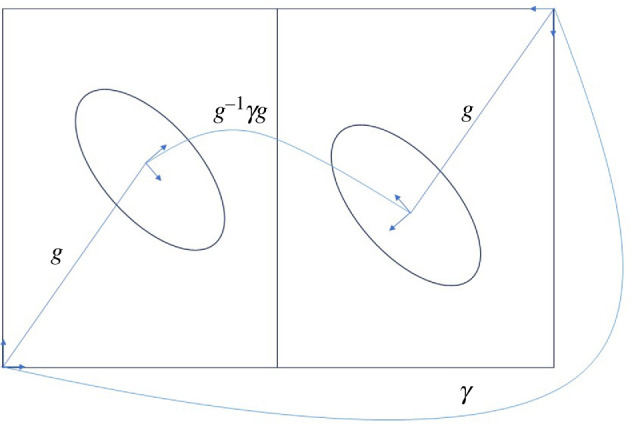
Conjugation constraints resulting in the left-hand side of (4.3) for the case of p2.

The left-hand side of (4.3) is illustrated for the case when Γ=p2 in figure 12. The full equation results by matching this with the kissing constraint, thus simultaneously bringing both ellipsoids into contact and preserving crystallographic symmetry.

The dimension of K is one dimension less than that of SE(n) . If i∈{1,...,m} in (4.3) then the total number of degrees of freedom of $\left(k_{1},...,k_{m}\right)$ becomes m⋅dim(K) , but as m increases, there comes a point where the number of constraints balances the number of degrees of freedom, and the values of $\left(k_{1},...,k_{m}\right)$ become fixed. At this point, it becomes possible to solve for g in a discrete subset of SE(n) . For this reason, it becomes important to understand conjugation constraints in SE(3) , which are discussed below.

Let,


$$X=\left(\begin{array}{cc} R_{X} & t_{X}\\ 0^{T} & 1 \end{array}\right)\;;\;\;\;A=\left(\begin{array}{cc} R_{A} & t_{A}\\ 0^{T} & 1 \end{array}\right)\;;\;\;\;B=\left(\begin{array}{cc} R_{B} & t_{B}\\ 0^{T} & 1 \end{array}\right)\;.$$

Then, the equation AX=XB or, equivalently,


(4.4) $$A=XBX^{-1}$$

can be written in terms of rotation and translation parts as


(4.5) $$R_{A}=R_{X}R_{B}R_{X}^{-1}$$

and


(4.6) $$t_{A}=(I-R_{X}R_{B}R_{X}^{T})t_{X}+R_{X}t_{B}.$$

Given one instance of (4.4), a family of solutions for X exists if some constraints are placed on A and B . When multiple instances of (4.4) are presented, with $\left(A_{i},B_{i}\right)$ for i=1,…,m taking the place of (A,B) , then necessary and sufficient conditions for X to have a solution can be expressed as constraints on the whole set $\left\{\left(A_{i},B_{i}\right)\;|\;i=1,\ldots ,m\right\}$ . This will be demonstrated below for n=2 and n=3 .

## 5. Arrangements of kissing ellipses in two dimensions with wallpaper symmetry constraints

Consider an arrangement of ellipses in the plane that are arranged by crystallographic symmetry. The elliptical boundaries must be in contact with symmetry mates and their interiors must not intersect. This could represent a deploying planar mechanism or the voids in a metamaterial. The formulation below explains how to generate such arrangements.

### (a) Conjugation constraints

In the planar case, rotations are of the form


$$R\left(\theta \right)=\left(\begin{array}{cc} cos\theta & -sin\theta \\ sin\theta & cos\theta \end{array}\right),$$

and there is a bijective correspondence


R(θ)⟷θ∈[0,2π).

Moreover, these planar commute:


$$R(\theta_{1})R(\theta_{2})=R(\theta_{2})R(\theta_{1}).$$

As a consequence, if $\theta_{1}\in (0,2\pi )$ then


$$\left[I-R\left(\theta_{1}\right)\right]R\left(\theta_{2}\right)=R\left(\theta_{2}\right)\left[I-R\left(\theta_{1}\right)\right],$$

and


$${\left[I-R(\theta_{1})\right]}^{-1}R(\theta_{2})=R(\theta_{2}){\left[I-R(\theta_{1})\right]}^{-1}.$$

Furthermore, (4.5) simplifies to


(5.1) $$R_{A}=R_{B},\;\;\text{or}\;\;\theta_{A}=\theta_{B},$$

and (5.1) simplifies to


(5.2) $$t_{A}=(I-R_{B})t_{X}+R_{X}t_{B}.$$

A *pole* of a planar motion is a point that is fixed. That is, g⋅x=x . Pure translations have no poles, but for all other motions they are determined by the equation


$$x={\left(I-R\right)}^{-1}t.$$

Given $\left(A_{1},B_{1}\right)$ and $\left(A_{2},B_{2}\right)$ with $\theta_{A_{i}}\neq 0\neq \theta_{B_{i}}$ , the necessary and sufficient conditions for a unique solution for X in the planar case are that


$$\theta_{A_{i}}=\theta_{B_{i}}$$

and that the distance between the poles of the A ’s is the same as that of the B ’s:


$$\left\|{\left(I-R_{A_{1}}\right)}^{-1}t_{A_{1}}-{\left(I-R_{A_{2}}\right)}^{-1}t_{A_{2}}\|=\|{\left(I-R_{B_{1}}\right)}^{-1}t_{B_{1}}-{\left(I-R_{B_{2}}\right)}^{-1}t_{B_{2}}\right\|$$

where $\|x\|=\sqrt{x\cdot x}.$

In this case, the solution procedure is straightforward: use (5.2) to isolate $t_{X}$ , then for i=1,2 write


$${\left(I-R_{B_{1}}\right)}^{-1}(t_{A_{1}}-R_{X}t_{B_{1}})={\left(I-R_{B_{2}}\right)}^{-1}(t_{A_{2}}-R_{X}t_{B_{2}}).$$

(Both sides of this equation are equal to $t_{X}$ .) This results in an equation of the form


$$R_{X}v=w$$

where ‖v‖=‖w‖ , which uniquely defines $R_{X}$ . Then back substitute to find $t_{X}$ .

Alternatively, if A is a pure translation, then so is B , and


(5.3) $$R_{A}=R_{B}=I,\;\;\text{or}\;\;\theta_{A}=\theta_{B}=0.$$

As a consequence, (5.3) simplifies to


(5.4) $$t_{A}=R_{X}t_{B}.$$

Given two such constraints, we can write


$$[t_{A_{1}},t_{A_{2}}]=R_{X}[t_{B_{1}},t_{B_{2}}],$$

and when $t_{A_{1}}\neq \alpha t_{A_{2}}$ for some α∈ℝ ,


(5.5) $$R_{X}=[t_{A_{1}},t_{A_{2}}]{\left[t_{B_{1}},t_{B_{2}}\right]}^{-1}.$$

There are no constraints on $t_{X}$ , but without loss of generality we can set $t_{X}≐0$ .

The mixed case when $R_{A_{1}}=R_{B_{1}}=I$ but $R_{A_{2}}=R_{B_{2}}\neq I$ , and hence,


$$t_{A_{1}}=R_{X}t_{B_{1}}\;\;\;\text{and}\;\;\;t_{A_{2}}=(I-R_{B_{2}})t_{X}+R_{X}t_{B_{2}}$$

will be addressed in future work.

### (b) The case of p1

In this case, space group elements are of the form $\gamma_{i}=(I,a_{i})$ , where $a_{1}$ and $a_{2}$ are the basis vectors for the lattice. To be a solid in the plane, there must be at least two independent contacts:


(5.6) $$g^{-1}\circ \gamma_{1}\circ g=k_{1}\;\;\;\text{and}\;\;\;g^{-1}\circ \gamma_{2}\circ g=k_{2}.$$

But these constraints mean that only the slice of kissing space corresponding to $\theta_{k_{i}}=0$ needs to be considered. Hence, only elements of the kissing space of the form $k_{i}=(I,t_{k_{i}})$ are allowed, where $t_{k_{i}}\in \partial (B+B)$ . This boundary is simply the boundary ∂B scaled by a factor of 2 . The general formula for the Minkowski sum $B+(-R_{k}B)$ reduces to B+B in this case because $R_{k}=I$ and B=−B for ellipsoids. In the case of more complicated space groups, where it is possible for $R_{k}\neq I$ , closed-form Minkowski sums of ellipsoids still exist [98].

Explicitly, this means that (5.5) becomes


$$R_{g}=[a_{1},a_{2}]{\left[t_{k_{1}},t_{k_{2}}\right]}^{-1}.$$

We could search the two-dimensional space defined by the variables $t_{k_{1}}$ and $t_{k_{2}}$ , each of which traces out a one-dimensional curve in the plane, until we were able to find a result which is a rotation matrix. But what is better is to enforce the constraint


$$R_{g}^{T}R_{g}=I,$$

which gives


(5.7) $${\left[t_{k_{1}},t_{k_{2}}\right]}^{T}[t_{k_{1}},t_{k_{2}}]={\left[a_{1},a_{2}\right]}^{T}[a_{1},a_{2}].$$

Note that the term on the right is the metric tensor for ${\mathbb{R}}^{2}$ expressed in the potentially non-orthogonal basis. And the determinant of both sides then gives


(5.8) $$|\;det\left[t_{k_{1}},t_{k_{2}}\right]\;|=V,$$

where V is the volume of the unit cell.

In the case when ∂B is an ellipse parameterized as


$$x\left(\phi_{i}\right)={\left[acos\phi_{i},bsin\phi_{i}\right]}^{T},$$

then ∂(B+B) is an ellipse parameterized as


$$t_{k_{i}}(\phi_{i})={\left[2a\cos\phi_{i},2b\sin\phi_{i}\right]}^{T}.$$

Substituting this into (5.7) gives equations that can be solved simultaneously to find $\phi_{1}$ and $\phi_{2}$ , and hence $R_{g}$ resulting in a packing.

Alternatively, substituting into (5.8) gives


$$4absin\left(\phi_{2}-\phi_{1}\right)=V,$$

from which we can write


$$\phi_{2}=\sin^{-1}\left(\frac{V}{4ab}\right)+\phi_{1}.$$

This can then be substituted back into any one of the entries of the matrix equation (5.7), and solved as a quadratic equation using the half-angle tangent formulas


$$\cos\phi =\frac{1-t^{2}}{1+t^{2}}\;\;\;\text{and}\;\;\;\sin\phi =\frac{2t}{1+t^{2}}\;.$$

## 6. Detailed calculations for arrangements of kissing ellipsoids in three dimensions with crystallographic symmetry constraints

Similar to the two-dimensional case, a deployable structure composed of ellipsoidal links or metamaterial consisting of ellipsoidal voids will have boundaries that kiss without intersecting interiors. This again amounts to conjugation constraints, and the use of invariants of the Special Euclidean Group.

### (a) Conjugation constraints

In three dimensions, rotations do not commute. By Euler’s Theorem, it is possible to write any non-trivial rotation as

,$$R_{X}=\exp(\theta_{X}{\hat{n}}_{X})$$

where $\theta_{X}\in (0,\pi )$ with $n_{X}\in S^{2}$ denoting the unit vector pointing along the axis of rotation, and ${\hat{n}}_{X}$ is the skew-symmetric matrix such that for any $y\in {\mathbb{R}}^{3}$ the equality ${\hat{n}}_{X}y=n_{X}\times y$ holds where × is the usual vector product.

The necessary and sufficient conditions for a unique solution to exist when given two instances of (4.4), $A_{1}X=XB_{1}$ and $A_{2}X=XB_{2}$ , when $R_{A_{i}}$ and $R_{B_{i}}$ are all non-degenerate were worked out in [99].

In contrast, when considering crystal packing, we often encounter the degenerate case where $\theta_{A_{i}}=\theta_{B_{i}}=0$ . From (4.5), $\text{tr}(R_{A_{i}})=\text{tr}(R_{B_{i}})$ , and so


$$R_{A_{i}}=I⟷R_{B_{i}}=I.$$

Then (5.1) reduces to


$$t_{A_{i}}=R_{X}t_{B_{i}},$$

and $t_{X}$ is arbitrary. If only two linearly independent translations for i=1,2 are presented then we can compute $R_{X}$ by solving


$$\left[t_{A_{1}},t_{A_{2}},t_{A_{1}}\times t_{A_{2}}\right]=R_{X}\left[t_{B_{1}},t_{B_{2}},t_{B_{1}}\times t_{B_{2}}\right],$$

Alternatively, three independent translations are presented, then we can solve for


$$\left[t_{A_{1}},t_{A_{2}},t_{A_{3}}\right]=R_{X}\left[t_{B_{1}},t_{B_{2}},t_{B_{3}}\right],$$

### (b) The case of P1

In this case, space group elements are of the form $\gamma_{i}=(I,a_{i})$ , where $a_{1}$ , $a_{2}$ and $a_{3}$ are the basis vectors for the lattice. To be a solid in three dimensions, there must be at least three independent contacts:


(6.1) $$g^{-1}\circ \gamma_{1}\circ g=k_{1}\;;\;\;\;g^{-1}\circ \gamma_{2}\circ g=k_{2}\;;\;\;\;\text{and}\;\;\;g^{-1}\circ \gamma_{3}\circ g=k_{3}.$$

But these constraints mean that only the slice of kissing space corresponding to $\theta_{k_{i}}=0$ needs to be considered. Hence, only elements of the kissing space of the form $k_{i}=(I,t_{k_{i}})$ are allowed, where $t_{k_{i}}\in \partial (B+B)$ . This boundary is simply the boundary ∂B scaled by a factor of 2 . From here things progress in the same way as in p1 , just with 3×3 matrices instead of 2×2 .

## 7. Conclusion

Group theory has been used here as a tool to reduce symmetrical deploying structures and metamaterials to their most fundamental irreducible components. In doing so, the design of dazzlingly complex looking structures becomes much more manageable. A theory for generating arrangements/configurations of bodies (or voids) in point contact is developed to produce feasible candidate structures. It is shown that such constraints involve the conjugation of crystallographic symmetry group elements by elements in the group of Euclidean motions. It is shown that the theory of Euclidean-group invariants together with methods for solving simultaneous polynomial equations then provides a tool for enumerating the designs of potential deployable structures and metamaterials. Future work will focus on applying this methodology to produce functional prototypes.

## Data Availability

This article has no additional data.

## Footnotes

^1^
The congruence symbol ‘ ≅ ’ is used instead of equality because the entities are equivalent abstractly in their behaviour, but on the left side, the elements are cosets in a coset space and on the right the elements are matrices, so as concrete sets they are different. In general, when two groups have such a relationship, they are called *isomorphic*.
